# Recycled Materials for Concrete and Other Composites

**DOI:** 10.3390/ma14092279

**Published:** 2021-04-28

**Authors:** Malgorzata Ulewicz

**Affiliations:** Faculty of Civil Engineering, Czestochowa University of Technology, Dabrowskiego 69 Street, PL 42-201 Czestochowa, Poland; malgorzata.ulewicz@pcz.pl; Tel.: +48-343-250-935

In recent years, industry, including the construction sector, has been focused on effectively reducing the consumption of natural resources, in compliance with the idea of sustainable development. The definition of “sustainable development” from the report “Our Common Future”, published in April 1987 by the World Commission on Environment and Development, defines sustainable development as “development that meets the needs of the present without compromising the ability of future generations to meet their own needs”. Therefore, the design and production of materials, including building materials, should be carried out with due regard to environmental protection requirements and with future generations in mind. It is particularly important to reduce the consumption of natural resources in the production of materials such as these, by using the latest technology, which can be replaced by waste materials and recycled building materials from the construction sector or waste materials from other industries.

Within this Special Issue of “Recycled materials for concrete and other composites”, there are published the latest results of scientific research on the use of recycled materials and post-production waste in the production of concrete and other composite materials. Research in this field is being carried out in many centers and universities around the world, including the USA, Colombia, Poland, Turkey, Jordan, Japan, China, India, Italy, Bangladesh, Spain, Korea, Mexico, Greece, Sweden, France, Qatar and South Africa.

This issue currently contains twenty-one original research papers and two review articles. The subject areas of these articles cover many aspects, including the production of composite materials containing recycled materials, their physicochemical and mechanical properties, corrosion resistance, elution of ions and their interactions with plasticizers or superplasticizers. Both interesting and practical solutions and the results of laboratory tests are presented. Irshidat and Al-Nuaimi [1] present the effect of utilizing carbon dust, generated as an industrial waste from aluminum factories, in the production of cementitious composites. Owsiak et al. [2] determine the physical properties of a three-component mineral binder containing hydrated lime, and cement bypass dust as a by-product derived from cement production. An interesting solution for the use of waste glass in composite products, including sand–lime, is described by Borek et al. [3]. Ternary mixtures of lime, sand and recycled waste glass had a higher compressive strength and lower density compared to the control sample, and the increase in the parameters was proportional to the amount of the replacement in these mixtures. Glass waste was also used by Jing et al. [4] as a fine aggregate in architectural mortar, while Wang et al. [5] investigated the micro-properties and mechanical properties of strain hardening cementitious composites containing recycled brick powder. It is worth paying attention to the work of Srimahachota et al. [6], which shows that recycled nylon fibers from waste fishing nets have great potential to be used as a strengthening fiber in cementitious material. Another way of using plastic waste is presented by Kane et al. [7] who have researched and examined the impact of biomineralization of plastic on the strength of plastic-reinforced mortar. In turn, Wang et al. [8] investigated the micro-properties and mechanical properties of cementitious composites containing sawdust and Liu et al. [9] present interface bonding behavior between the steel tube and the core concrete of a concrete-filled steel tube with circulating fluidized bed bottom ash.

A number of works in this Special Issue present the latest achievements in the synthesis of innovative concrete-containing recycled waste and materials and their properties. Bai et al. [10] determine the mechanical properties of recycled aggregate concrete under uniaxial compression based on a statistical damage model. Choi et al. [11] determine the compressive strength, chloride ion penetrability, and carbonation characteristics of concrete with both ferronickel slag and blast furnace slag. The physical and mechanical properties of concrete composites containing waste thermoplastic elastomer from the production process of car floor mats is presented by Ulewicz et al. [12]. Miah et al. [13] present the effect of steel slag aggregate as a substitute for conventionally used brick aggregate on the physical, mechanical and durability performances of concretes. In turn, Dobiszewska and Beycioğlu [14] show that waste basalt powder, which is a by-product of the production of mineral–asphalt mixtures, used as a partial sand replacement, increases the compressive strength of concretes. According to microstructural analyses, the presence of basalt powder in concrete mixes is beneficial for cement hydration products, and basalt powder-substituted concretes have lower porosity within the interfacial transition zone. The durability of concrete with recycled concrete aggregate (from precast concrete), treated by a coating of a cement paste dissociation agent, depends, as shown by Yang and Lee [15], on mixing methods. Additionally, Landa-Sánchez et al. [16] determine the corrosion resistance of green concrete (GC) admixtures containing recycled coarse aggregate and reinforced with AISI 1080 carbon steel and AISI 304 stainless steel. In turn, Król [17] presents the result of leaching of chromium from concretes made of Portland cement CEM I and slag cement CEM III/B containing 75% granulated blast furnace slag.

It is worth paying attention to the article by Ryms et al. [18] wherein the authors cover a new application for char as a carrier of phase-change materials (PCMs) used as an additive to building materials. Thus far, no one has tried to utilize pyrolytic biochar with a well-developed internal surface for permanent PCM adsorption. Gołaszewski et al. [19] present the influence of raw and ground calcareous fly ash on rheological properties and other effects of admixtures (plasticizers and superplasticizers), in particular, the amount of air in the mixture and the level of heat of hydration.

Recycled materials can also be successfully used as an additive to asphalt mixtures. Beycioğlu et al. [20] present the possibilities of using waste powder from glass fiber-reinforced polyester (GRP) pipes (GRP-WPs) for asphalt mixtures as a filler and Sánchez-Cotte et al. [21] present the implicational possibilities of using recycled concrete aggregate (recycled concrete aggregate of a building and recycled concrete aggregate from a pavement) as a replacement for natural aggregates in road construction. This research concludes that the studied recycled concrete aggregates might be used as replacements for coarse aggregate in asphalt mixtures since their chemical properties do not affect the overall chemical stability of the asphalt mixture.

This Special Issue is completed by a review article by Lavagna et al. [22]. This analytical mini-review presents the compression strength of rubberized concrete as a function of the amount of recycled tire crumb rubber. Glaydson dos Reis [23] discusses the generation and recycling of construction and demolition wastes (CDWs) as well as their main uses as raw materials for the construction engineering sector.

All the articles published in this Special Issue have been reviewed by recognized experts in the relevant fields of science. As Guest Editor, I would like to acknowledge all the authors for their valuable contributions. I would also like to thank the reviewers for their comments and suggestions that have greatly improved the quality of the papers. Last, but not least, I would also like to thank the Section Managing Editor, Ms. Freda Zhang, for her kind assistance in the preparation of this Special Issue of the journal.
